# Evaluating Doctor Performance: Ordinal Regression-Based Approach

**DOI:** 10.2196/jmir.9300

**Published:** 2018-07-18

**Authors:** Yong Shi, Peijia Li, Xiaodan Yu, Huadong Wang, Lingfeng Niu

**Affiliations:** ^1^ School of Economics and Management University of Chinese Academy of Sciences Beijing China; ^2^ Key Laboratory of Big Data Mining and Knowledge Management Chinese Academy of Sciences Beijing China; ^3^ Research Center on Fictitious Economy and Data Science Chinese Academy of Sciences Beijing China; ^4^ College of Information Science and Technology University of Nebraska at Omaha Omaha, NE United States; ^5^ School of Computer and Control Engineering University of Chinese Academy of Sciences Beijing China; ^6^ Development and Planning Research Institute Ningbo China; ^7^ School of Information Technology and Management University of International Business and Economics Beijing China

**Keywords:** performance evaluation, ordinal regression, mHealth, support vector machines, ordinal partitioning

## Abstract

**Background:**

Doctor’s performance evaluation is an important task in mobile health (mHealth), which aims to evaluate the overall quality of online diagnosis and patient outcomes so that customer satisfaction and loyalty can be attained. However, most patients tend not to rate doctors’ performance, therefore, it is imperative to develop a model to make doctor’s performance evaluation automatic. When evaluating doctors’ performance, we rate it into a score label that is as close as possible to the true one.

**Objective:**

This study aims to perform automatic doctor’s performance evaluation from online textual consultations between doctors and patients by way of a novel machine learning method.

**Methods:**

We propose a solution that models doctor’s performance evaluation as an ordinal regression problem. In doing so, a support vector machine combined with an ordinal partitioning model (SVMOP), along with an innovative predictive function will be developed to capture the hidden preferences of the ordering labels over doctor’s performance evaluation. When engineering the basic text features, eight customized features (extracted from over 70,000 medical entries) were added and further boosted by the Gradient Boosting Decision Tree algorithm.

**Results:**

Real data sets from one of the largest mobile doctor/patient communication platforms in China are used in our study. Statistically, 64% of data on mHealth platforms lack the evaluation labels from patients. Experimental results reveal that our approach can support an automatic doctor performance evaluation. Compared with other auto-evaluation models, SVMOP improves mean absolute error (MAE) by 0.1, mean square error (MSE) by 0.5, pairwise accuracy (PAcc) by 5%; the suggested customized features improve MAE by 0.1, MSE by 0.2, PAcc by 3%. After boosting, performance is further improved. Based on SVMOP, predictive features like politeness and sentiment words can be mined, which can be further applied to guide the development of mHealth platforms.

**Conclusions:**

The initial modelling of doctor performance evaluation is an ordinal regression problem. Experiments show that the performance of our proposed model with revised prediction function is better than many other machine learning methods on MAE, MSE, as well as PAcc. With this model, the mHealth platform could not only make an online auto-evaluation of physician performance, but also obtain the most effective features, thereby guiding physician performance and the development of mHealth platforms.

## Introduction

With the advancement of the internet and electronic devices, mobile heath (mHealth), is defined by the World Health Organization as “medical and public health practice supported by mobile devices,” is becoming increasingly popular. mHealth has strong links with electronic health [1] with some differences [2]. According to an mHealth survey [3], 80% of physicians use smartphones and medical apps and 61% of people have downloaded a medical app. Meanwhile, 93% of physicians believe that mHealth apps can help to improve patients’ health. The doctor/patient communication platform is one of the most common areas in mHealth, for example, “Dermatologist-on-Call” in America and, in China, “Chunyu-Doctor-online” and “Good-Doctor-online.” These platforms digitally connect doctors and patients and offer a convenient channel for doctor/patient communication and help doctors use time more efficiently. Additionally, the mHealth platforms are more beneficial to under-developed countries, especially when medical resources are scarce, and quality medical care is difficult to access.

Many doctor/patient communication platforms face the challenge of how to evaluate the performance of doctors online. Doctor performance evaluation serves to increase the probability for patients to have a positive experience and improve patient satisfaction [4-6]. Meanwhile, doctor performance evaluation also helps doctors to improve medical practice [7]. In this paper, we address the issue of doctor performance evaluation (DPE).

Various methods have been attempted that address the issue of DPEs. Ratings by patients is the most common method, which averages patient ratings when evaluating physicians. Physician ratings are usually based on the following labels: (1) very unsatisfied, (2) unsatisfied, (3) neutral, (4) satisfied, and (5) very satisfied. Statistics show that only a small proportion of patients rate their doctors on mHealth platforms, and in China, only 36% of patients rated their doctor at the end of the consultation.

A physician expert assesses the doctors’ professional skills and services. In this combined method, experts re-rate the patient’s unsatisfied consultations and judge whether the doctors are qualified. It is an advanced evaluation method, which not only considers patient ratings but also imbues prior professional knowledge. Therefore, this method is recommended but heavily depends on the patient’s ratings.

Considering the amount of data generated from doctor/patient communication platforms every day, machine learning techniques are recommended. In the machine learning area, some scholars have rated patient satisfaction into standard classification algorithms [8,9] but ignore the ordering information between labels. The ordering information between labels as mentioned above are, (1) very unsatisfied, (2) unsatisfied, (3) neutral, (4) satisfied, and (5) very satisfied. The label “unsatisfied” is adjacent to the label “very unsatisfied” and the label “neutral,” while the label “very unsatisfied” is not next to the label “neutral,” therefore the ordering of information is extremely important. Rating a “very unsatisfied” doctor consultation as an “unsatisfied” one is less of an error than rating it as “very satisfied.” Therefore, it is important that the predicted labels are not only accurate but as close to the true labels as possible. This method of classifying the instances into the nearest ordinal labels, is called ordinal regression (OR) [10,11], and the overall evaluation model is ordinal regression for doctor performance evaluation (OR-DPE).

In supervised learning OR resides between multi-classification and metric regression. The difference between the two is that the labels of the latter are in a limited but unordered set. The difference between OR and metric regression is that the OR labels do not represent numerical values. Although standard multi-classification and regression algorithms can be used to solve OR problems, they ignore the ordering information between labels. Some researchers [11,12] have proved that ordering information benefits modelling greatly. There exist many models especially designed for OR. The “Proportional Odds” model, designed in 1980 [13], is one of the earliest such models. Since then a wide range of OR models have been proposed including support vector machine (SVM)-based models [10,14-17], Neural Network-based models [18], Gaussian Process models [19], and more. An excellent survey [11], provides a comprehensive literature review about OR. The SVM-based model is one of the most popular models used in the field.

**Figure 1:**
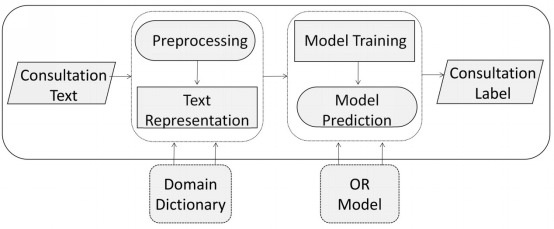
The general workflow of the ordinal regression for doctor performance evaluation (OR-DPE) model.

In the OR-DPE model, the consultation text is the input, and one label from the set of (1) very unsatisfied, (2) unsatisfied, (3) neutral, (4) satisfied, and (5) very satisfied is the output. The workflow of the OR-DPE model is shown in Figure 1. OR-DPE comprises of text preprocessing, representation, model training, and predictability. Because the communication between doctor and patient is through a text message, the DPE task is like text mining. The consultation texts are preprocessed and displayed as high dimensional vectors. Because the SVM-based model with linear kernel [14] performs excellently on large-scale data and is well suited for text mining fields, this model is preferred to address the DPE. In this paper, a new SVM-based Ordinal Partitioning model (SVMOP) is proposed as the OR model for DPE. With the SVMOP model, OR-DPE can, not only make sure that the predicted labels are as correct as possible, but also ensure that the incorrect labels are as close to true as possible. To our knowledge, this is the first time that the issue of DPE has been conceptualized as an ordinal regression task. Empirical studies on real data sets from one of the largest mobile doctor/patient communication platforms in China show that the model can achieve state-of-the-art performance from multiple metrics.

## Methods

### Preprocessing and Text Representation

The original corpus should be preprocessed, and each sample should be represented as an input vector. In the preprocessing step, punctuation and stop words will be removed. If the experimental data is written in Chinese, the words must be segmented as in Chinese text. Sentences are represented as character strings without natural delimiters. Chinese Word Segmentation (CWS) is used to identify word sequences in a sentence and mark boundaries in appropriate places. For example, CWS can put the character sequence “天花” together as a Chinese word for “smallpox” rather than the individual Chinese character “天” (sky) and “花” (flower) respectively. Word segmentation is a preliminary and important step for preprocessing. Most methods take the CWS as a sequence labeling problem [20], which can be formalized as supervised learning methods with customized features. Additionally, domain dictionaries with technical terms as ancillary resources, are beneficial for CWS and medical feature extraction. Here, 3 medical dictionaries are employed; one for Illness, one for Symptoms and one for Medicine. Most terms in the dictionaries are customized by medical experts and extended with new word detection techniques. We have collected 49,758 illness and symptom terms and 24,975 medical terms. Information about the dictionaries are shown in Table 1. For this purpose, we combined the dictionaries with Jieba tool, an open sourced Chinese segmentation software, for word segmentation.

For text representation, each sample is represented as an input vector where each dimension of the vector represents a feature. The element is the corresponding feature value. Feature engineering plays an important role in text mining. Apart from the basic text features such as Bag of Words (BOW) [21], unigrams, and bigrams, the custom medical features that can mirror some characteristics of the platform are utilized. These are specifically designed for the doctor/patient communication platform by domain experts and most are based on medical dictionaries. Typical text and medical features used in OR-DPE are presented in Table 2. Customized features (F1-F8) can capture domain knowledge: the count of medicine and symptom names in doctors’ answers reflects the doctors’ professional level; the number of Chinese characters in doctors’ answers mirrors the service attitudes, and more. Likewise, the text features (F9 and F10) cover most consultation information. The feature value is the numerical value of the feature while the feature value of text features is the term frequency inverted document frequency (TF-IDF) [22]. TF-IDF reflects how important a word is to a document. If a word occurs rarely but appears frequently in a sample, it is most likely to reflect the characteristics of this sample. Specifically, TF-IDF is the product of two statistics: term frequency and inverse document frequency, where the former represents the frequency and the latter represents the inverse frequency of occurrence in all samples.

**Table 1:** The details about the medical dictionaries. “1≤terms≤3” means the number of terms having a character length less than 3 but greater than 1.

Number of phrases	Dictionary Name	
	Illness and Symptom Dictionary (N=49,758)	Medicine Dictionary (N=24,975)
1≤terms≤3, n (%)	32840 (66.00)	3746 (15.00)
4≤terms≤6, n (%)	16918 (34.00)	14486 (58.00)
terms≥7, n (%)	0 (0)	6743 (27.00)
Representative examples	神经衰弱症 (Neurosis), 高血压 (HTN), 天花 (smallpox)	帕罗西汀 (Paroxetine), 盐酸环苯扎林 (Flexeril)

**Table 2:** F1-F8 represent the customized medical features, while F9 and F10 are the text features.

Feature	Description
F1	The number of symptom names in doctors’ answers
F2	The number of illness names in doctors’ answers
F3	The number of medicine names in doctors’ answers
F4	The number of patients’ questions
F5	The number of doctors’ answers
F6	The response time for the patient’s first question
F7	The number of Chinese characters in patients’ questions
F8	The number of Chinese characters in doctors’ answers
F9	Unigrams
F10	Bigrams

The quantity of text features is so large that the customized features (see Table 2) can easily be overshadowed. To highlight the importance of customized features, they are boosted by the Gradient Boosting Decision Tree (GBDT) [23]. GBDT is a powerful tool in many industrial communities [24]. GBDT mines the most effective features and feature combinations by a decision tree to boost the performance of regression and classification tasks. This technique is applied to increase the number of custom medical feature combinations. The main idea of GBDT is to combine weak learners into a single, strong learner like other boosting methods. GBDT is an iteration algorithm, which is composed of multiple decision trees. In the *m*-th iteration of GBDT, assumes that there are some imperfect models, *F*_m_. The GBDT would construct a better model *F*_m+1_ to approach the best model by adding an estimator *h*, namely *F*_m+1_ = *F*_m_*(x)* + *h(x).* Then the problem is transformed by the question of how to find *h(x)*. As the above equations imply, a perfect *h* should satisfy the equation:


*h(x)* =
*F*_m+1_ –
*F*_m_
*(x)* ≈
*y – F*_m_
*(x)*

where *y* is the true label, *y – F*_m_*(x)* is called a loss function. In practice, a general way is to apply square loss function is: ½(*y* –*F*_m_*(x))*^2^. Because the residual is exactly the negative gradients of the squared loss function. The problem on the left can then be solved directly by gradient descent algorithms. In our work, we apply GBDT to boost the 8 customized features shown in Table 2 to generate several effective feature combinations. According to the statistics, the number of features is 363,336 with text features, and 363,344 if adding the 8 customized features. After boosting the customized features, the number becomes 370,858. Another 7514 combined customized feature combinations have been added. The performances of various features are shown in Section Results.

### Model Training

#### How the Ordinal Regression Method for the Ordinal Regression for Doctor Performance Evaluation Model Was Chosen

There are many different models of OR. Referring to an OR survey [11], the models are grouped into three categories, namely the (1) naive approach, (2) threshold approach, and (3) ordinal partitioning approach. These models have corresponding strengths and weakness. The naive approach considers OR naively, as a standard classification task or a regression task [14,25]. At the same time, the ordering information between labels has been ignored. The threshold approach is based on the idea of approximating a real value predictor and then dividing the real line into intervals [10,15,26,27]. Assuming *P* is the number of categories, the objective of threshold-based OR models is to seek *P* –1 parallel hyperplanes further dividing the data into ordered classes. The ordinal partitioning approach uses the ordering information to decompose the ordinal regression into several binary classification tasks. For binary classification, there are many models to choose from. For example, Frank and Hall [16], applied decision trees as submodels while Waegeman and Boullart [17] used weighted SVMs as binary classifiers.

Since the ordering of information is conducive to model building [11], we chose the OR model from the latter two methods. As the number of samples is large and the dimension of the representative vectors is high, a model was chosen that can handle large-scale and high dimensional data. So, the ordinal partitioning approach is used instead of the threshold approach for OR problems depending on paralleled hyperplanes. There are many binary classifiers that can be chosen from the submodels. Hsieh et al [14] showed that the linear SVM is a robust tool that can deal with large-scale and high dimensional data. Inspired by these, we want to combine SVM with Ordinal Partitioning (SVMOP) as the OR model for the OR-DPE.

**Figure 2:**
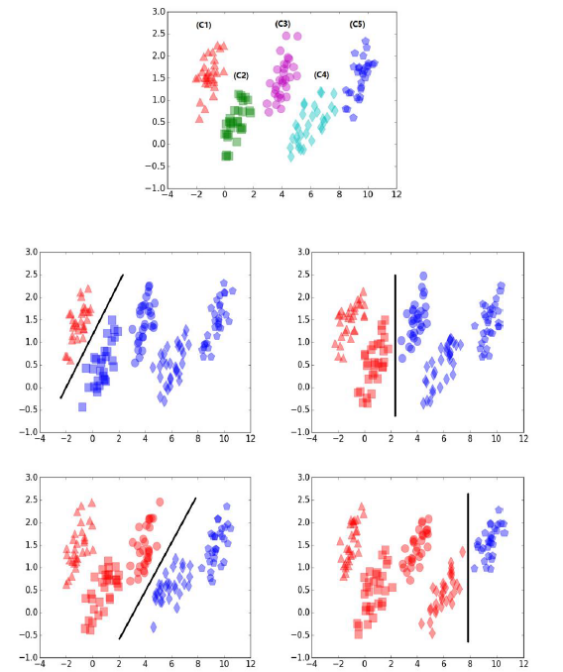
The demo that shows how a combined support vector machine and ordinal partitioning scheme model (SVMOP) works on ordinal data.

#### SVMOP Model and Training Algorithm

The OR problem can be described as follows: given a training set 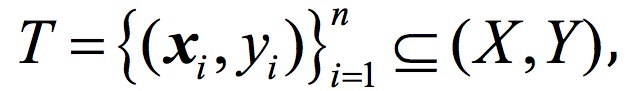
where *x* ϵ *R*^l^ is the *i*-th input vector (*i*=1,2,…,n), where *n* is the number of instances, *l* is the number of features, and *y_i_* ϵ *Y*_i_ is the label of *x*_i_. Assuming there are *P* categories and without loss of generality, we take the label set *Y*={1,2,…P}. The goal of OR is to find a function ƒ: *X* → *Y* to predict the label of a new instance *x*. As mentioned earlier, SVMOP will be embedded into the OR-DPE model. Figure 2 illustrates the SVMOP procedure. In this figure, five ordinal categories of data are represented by different colors and shapes. The idea of SVMOP is to partition the overall model into *P* –1 binary classifications. Then the associated question: “Is the rank of the input greater than p?” can be asked. Here *p*=1,2,…,*P* –1. Therefore, the rank of *x* can be determined by a sequence of these binary classification problems. Specifically, when training the *p*-th binary classifier, the label *y*_i_ is retransformed to a new class label depending on whether the label ŷ_pi_ is greater than *p* or not, namely:

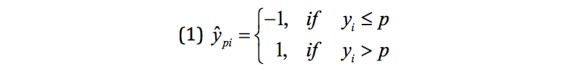



where *i*=1,2,…,*n*. Therefore, the problem can be reformulated: given a training set 
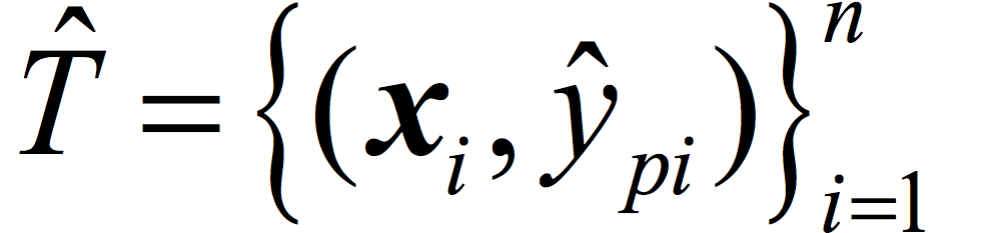
, where *x* ϵ *R*^l^ is the *i*-th input sample, ŷ_pi_ ϵ{-1,1} is defined by equation 1. The model aims to find a function to predict the ordered labels of new instances.

Linear SVM is one of the best candidates among the binary classifiers dealing with high dimensional data. Then linear SVM is taken as the *p*-th sub-model:

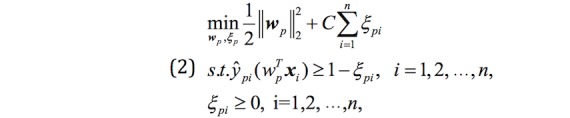



Where *w*_p_ represents the parameter of the *p*-th submodel, ξ_pi_ is the slack variable of the *p*-th submodel. As for the optimization solver, we chose the Dual Coordinate Descent algorithm (DCD) as the training algorithm of SVM [14]. DCD is one of the most effective training algorithms for linear SVMs. It solves the model in equation 2 by the Lagrange dual form. The dual form of the *p*-th sub-model in equation 2 is given as equation 3. Without loss of generality, we ignore the subscript p in the dual form:

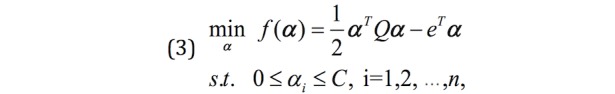



where 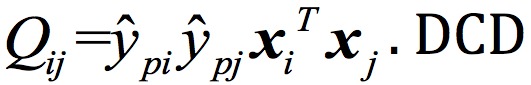
is to employ a classic divide-and-conquer method for optimizing high dimensional problems. It starts from an initial zero vector **α**^0^=**0** and generates a sequence of vectors 
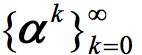
. For each iteration step, the algorithm sequentially selects one dimension associated with α to optimize by fixing other dimensions. Suppose α* is the solution of equation 3 then the optimal value of *w*_p_ for equation 2 can be computed as follows:

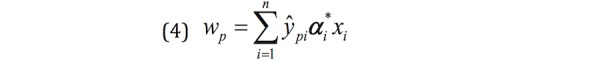



### Model Prediction

For model prediction, the research [11] shows that it is important to construct an effective rule for predicting new instances in the ordinal partitioning-based OR models. Many existing ways are based on the probability manipulation or outcomes by submodels to predict the label of a new instance. In the work by Frank et al [16], when estimating the probabilities for the first and the last class, the authors were dependent on a corresponding classifier. However, it needs to rely on two adjacent classifiers when computing the middle classes. This prediction method is simple and easy to implement, but may lead to a negative probability [11,28]. Another example in the work [17], the authors combined the outcomes of all the submodels to predict the label of a new instance *x*. However, their prediction function may cause ambiguities for some test samples.

To alleviate the problem with the above prediction functions, we propose a new prediction function as shown in equation 5:

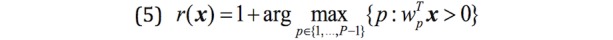



where *r(x* *)*=1 if none of *w*^T^_p_*x* is greater than 0. This prediction function relies on the discriminant planes and joins all binary classifiers to obtain a single classification. The *p*-th binary classifier provides the answer to the associated question: “Is the rank of the input *x* greater than *p*?”, where *p*=1,2,…,*P* –1. That is, for prediction, the new sample *x* would be asked by a sequence of the questions above. And last, the predicted label equals *r(x* *)* which represents the satisfaction degree. The greater *r(x* *)*, the more satisfied.

### Statistical Methods and Evaluation Metrics

To better highlight the characteristics of ordinal regression models, we evaluated the performance with the following three common evaluation measures: (1) mean absolute error (MAE) [10,11,29], (2) mean square error (MSE) [30,31,32], and (3) pairwise accuracy (PAcc) [29,33,34]. MAE and MSE can directly measure the degree of deviation between the true label (*gold*_i_) and predicted label (*predicted*). They can be defined by the following equations:

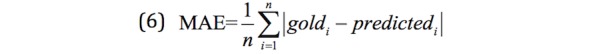

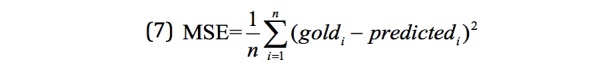





Since they are metrics measuring the error, the lower they are, the better their performance. PAcc is widely applied in the medical data analysis, ranking and statistics fields with the name of concordance index or Kendall τ [34,35]. PAcc could reflect the correct ratio of ranking between pairwise instances. Specifically, the set of preference evaluation pairs is represented as *S,S*={(*i,j*) | *gold_i_ > gold_j_* }.

The PAcc is given by

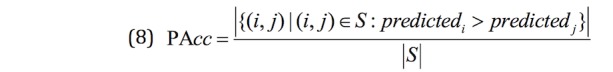



where “| *S* |” represents the number of the set *S*. It accords with the rule: the greater, the better.

### Mining Predictive Features

Apart from rating doctors’ performance, we continue to explore the most predictive features among text features and customized features in DPE. In general, predictive features always play significant and instructive roles on the platform construction. In this case, the most important features were extracted by analyzing the weight matrix *W ϵ R^l x (P-1))^*, where *l* and *P* –1 are the dimensions of the matrix. As mentioned, *l* is the total number of all the features (that is, *l*=363*,* 344) and *P* is the number of categories, where *P* –1 is the number of the submodels. In equation 2, *W* is composed of the weight parameters, with *w* in each submodel, namely *W*=(*w*_1_*,w*_2_*,…,w*_P–1_). We denote *W(j,:)* as the *j*-th row vector and the absolute value of the elements in the row vector represents the contributions to each submodel for the *j*-th feature. The larger the value is, the more predictive property the feature has. For every feature in each kind of text feature or customized feature, described in Table 2, it owns its corresponding weight vector *W(j,:)*, where 1 ≤ *j* ≤ *l*. We compute the total contribution Con_j_ of the *j*-th feature to the model decision by equation 9:

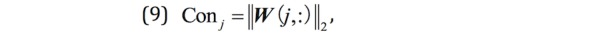



where “∥ ∥_2_” represents the L2-norm of a vector. When the contributions of all the features have been computed, they would be ranked and hence obtain the top-most predictive features.

## Results

### Preparation of Datasets

**Figure 3:**
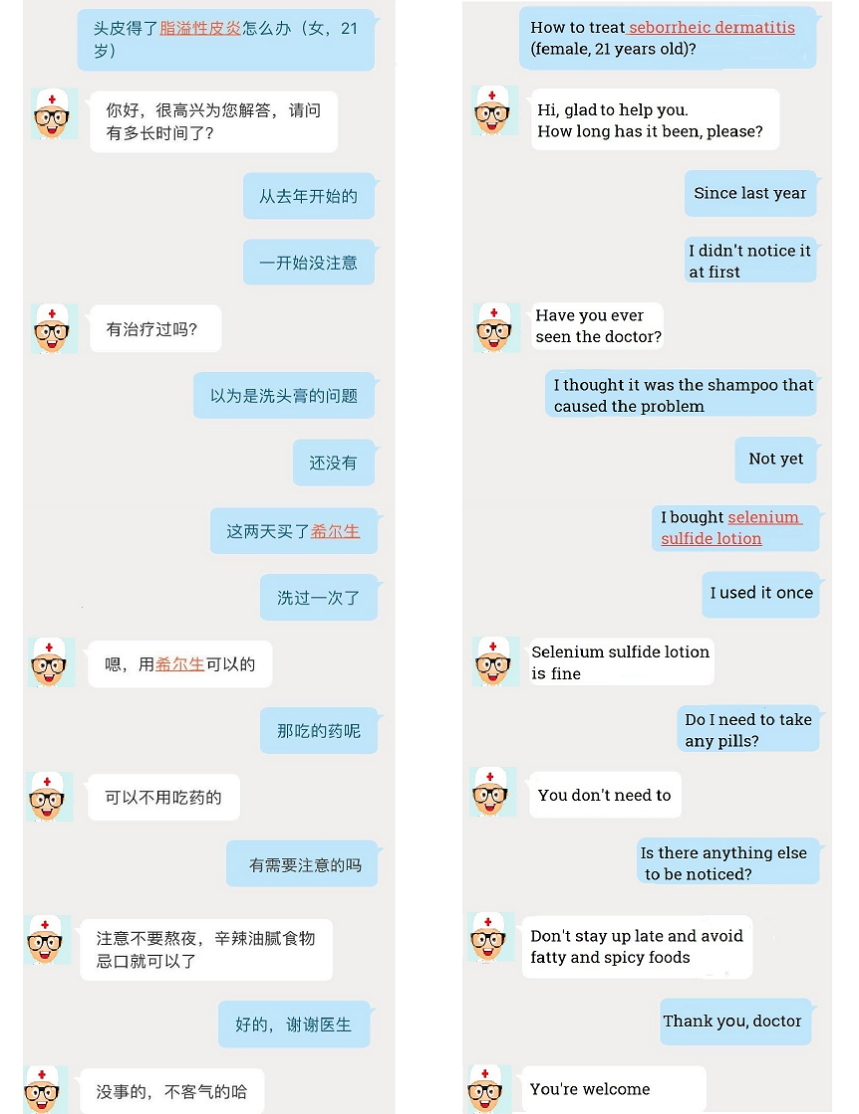
An example of consultation letters on Platform X. The left subfigure is the real consultation on Platform X by mobile software applications but without sensitive information such as doctors’ photos. The right one is the version in English.

To validate the proposed model on real data, the data from one of the popular doctor/patient consultation platforms (Platform X) in China was chosen as the experimental data sets. In practice, the platform maintains long-term cooperation with us. However, in order to comply with the confidentiality agreement with the platform, we are not able to use the real name of the platform in the paper but instead we use the name Platform X throughout. On Platform X, the consultation mainly consists of patients’ questions (eg, “医生您好，如何补钙” [Hi doctor, how can I add more calcium?]) and the response (eg, “很高兴为您服务，您可以通过牛奶，豆制品，鱼等食物，也可以口服碳酸钙和葡萄酸钙” [“Glad to help you,” “you can eat foods such as milk, bean products, and fish” and “You can also take calcium gluconate and calcium carbonate directly”]). To introduce experimental data, an actual consultation letter was used (Figure 3). Based on analysis of patients’ questions, multiple question types are proposed. Most questions are about ailments that are not serious or related to personal privacy, like chronic pharyngitis and dermatosis. And, because they are flexible and convenient, most consultations are done through mobile software applications. Platform X faces the same problem when evaluating doctor’s online performance. Platform X did not receive direct customer ratings or feedback since most patients tend to rate the very good or bad and at times feedback was not received because, for example, a customer may have been offline.

Of a sampling of 2,337,828 clinical data collected over the last twenty days, only 841,618 (36%) of the data was labeled by patients, which proves that most patients do not like to provide feedback. From the labeled data, only 720 instances, 1712 instances, and 8737 instances were labeled very unsatisfied, unsatisfied, and neutral respectively. The unbalanced data challenges the model. To alleviate the issue of unbalanced data and collect more instances of “very unsatisfied,” we chose sample training data from Platform X’s database. As previously mentioned, we have a long-term association with Platform X. It takes approximately two hours to access the entire database. Data collected is valid for about 18 months. After that, the same number of instances from each category are sampled. After filtering the data, (removing consultations with a length of less than 10 words), we have approximately 112,485 instances to use as experimental datasets. Each category contains approximately 22,497 samples which are randomly split into five sections with four sections serving as the training sets and the remaining one as the test set.

### Baseline Methods

To better reflect the effectiveness of the proposed model with the above metrics, the following baseline methods will be compared with our model. These methods are popular and representative in OR fields. To solve the high dimensional data efficiently, the following models all use the linear kernel. The DCD algorithm is adopted to solve the following models. These are implemented by modifying the open source package LIBLINEAR [36] directly and all the codes related to the experiment are uploaded to a Github website. The following methods were used to compare with our model.

1. SVC [14]: Support vector classification with one versus the rest. This model belongs to the naive approach.
2. SVR [37]: Support vector regression. The ordinal labels are treated as continuous values. When predicting new instances, the predictions for test instances are rounded to the nearest ordinal label and the model belongs to the naive approach.
3. LR [38]: Logistic regression one versus the rest. This model belongs to the naive approach.
4. SVOR [10]: Support vector ordinal regression. This model aims to optimize multiple thresholds to define parallel discriminant hyperplanes. The SVOR model is used with implicit constraints and belongs to the threshold approach.
5. RedSVM [39]: Reduction support vector machine. A threshold approach and it is a reduction framework from ordinal ranking to weighted binary classification by extending examples.

### Evaluation Performance

First, we compare the performance of five different baselines with our SVMOP model using different sets of features, including (1) text features (T), (2) text and customized features (T+C), and (3) text, customized, and boosted features (T+C+B). Three metrics, (ie, MAE, MSE, and PAcc) are used to evaluate model performance. Table 3 shows the results of the experiment. The best performance for each metric is represented by the footnote “k” while the best “one of” feature sets is represented by the footnote “e.” In Table 3, the SVMOP model outperforms other baselines on MAE, MSE and PAcc with each type of feature sets, demonstrating the effectiveness of our model. On different set of features, all models achieved better performance with feature set T+C and feature set T+C+B. Furthermore, compared with feature set T+C+B, feature set T+C attained more improvement. In other words, using customized features are important for performance improvement

**Table 3:** Performances of various models having multiple feature sets (T, T+C, T+C+B) are shown in this table.

Method	Text (T)			Text and Customized (T+C)			Text, Customized, and Booster (T+C+B)			
	MAE^a^	MSE^b^	PAcc^c^ (%)	MAE	MSE	PAcc (%)		MAE	MSE	PAcc (%)
SVC^d^	0.7925	1.7613	53.32	0.6726	1.3759	57.32		0.6212^e^	1.1981^e^	59.05^e^
SVR^f^	0.8023	1.3302	49.74	0.7050	1.1106	54.24		0.6906^e^	1.0332^e^	56.37^e^
LR^g^	0.7716	1.6883	53.86	0.6359	1.2606	57.77		0.5978^e^	1.1310^e^	59.50^e^
SVOR^h^	0.8086	1.3742	49.58	0.7170	1.1167	54.09		0.6665^e^	1.0143^e^	57.20^e^
RedSVM^i^	0.8046	1.3715	50.11	0.7168	1.1127	54.00		0.6718^e^	1.0236^e^	57.21^e^
SVMOP^j^	0.7054^k^	1.2706^k^	54.11^k^	0.6130^k^	1.0108^k^	57.92^k^		0.5864^e,k^	0.9605^e,k^	59.65^e,k^

^a^MAE: mean absolute error.

^b^MSE: mean standard error.

^c^PAcc: pairwise accuracy.

^d^SVC: support vector classification.

^e^Best “one of” feature sets.

^f^SVR: support vector regression.

^g^LR: logistic regression.

^h^SVOR: support vector ordinal regression.

^i^RedSVM: reduction support vector machine.

^j^SVMOP: a combined support vector machine and ordinal partitioning scheme model.

^k^Best performance for each metric.

**Figure 4:**
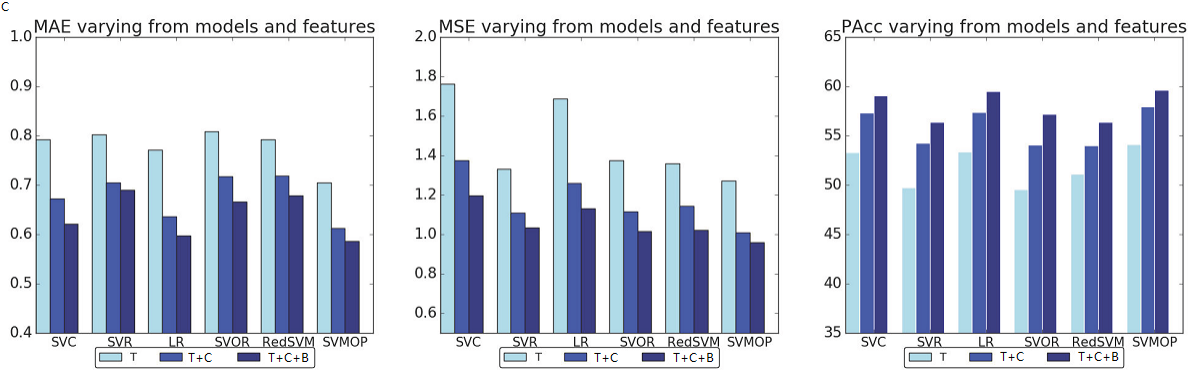
Mean absolute error (MAE), mean square error (MSE), and pairwise accuracy (PAcc) varying from different models and different feature sets. LR: logistic regression; RedSVM: reduction support vector machine; SVC: support vector classification; SVMOP: a combined support vector machine and ordinal partitioning scheme model; SVOR: support vector ordinal regression; T: text features; T+C: text and customized features; T+C+B: text, customized, and boosted features.

**Figure 5:**
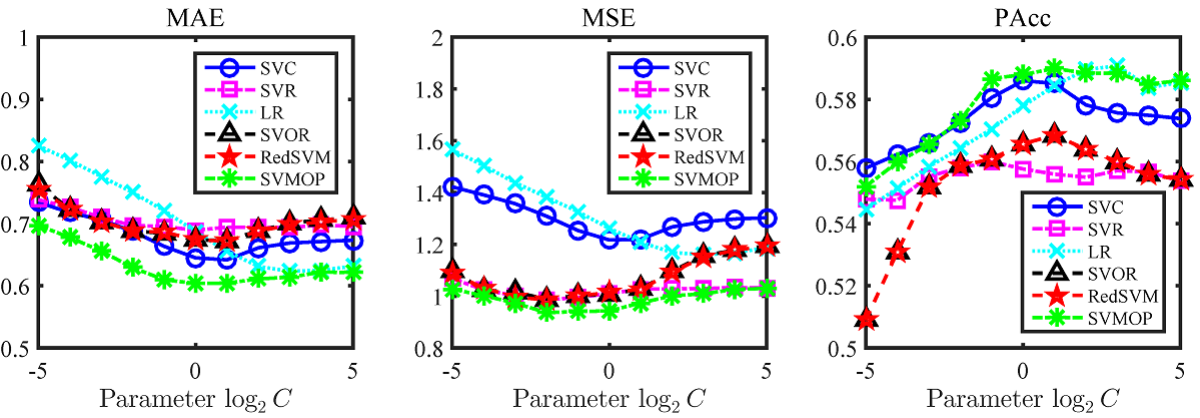
The different performances with different parameters in training process with the text, customized, and boosted feature set (T+C+B). LR: logistic regression; MAE: mean absolute error; MSE: mean square error; PAcc: pairwise accuracy; RedSVM: reduction support vector machine; SVC: support vector classification; SVMOP: a combined support vector machine and ordinal partitioning scheme model; SVOR: support vector ordinal regression; SVR: support vector regression.

Figure 4 displays the performances of 6 models on 3 measures, namely MAS, MSE, and PAcc. As we can see, SVMOP greatly outperforms the other models on MAE, MSE, and PAcc. Additionally, the models that consider ordering information, namely, SVOR, RedSVM, and SVMOP, perform better than the rest on MSE; and SVC and LR achieve comparable performances with SVMOP on PAcc. To investigate the influence of the parameter, we show the various performance of each model as we change the parameter log_2_*C* in a range [-5,5]. In Figure 5, we find that the performances vary as the parameters change and the model can achieve the best performance in this range.

**Figure 6:**
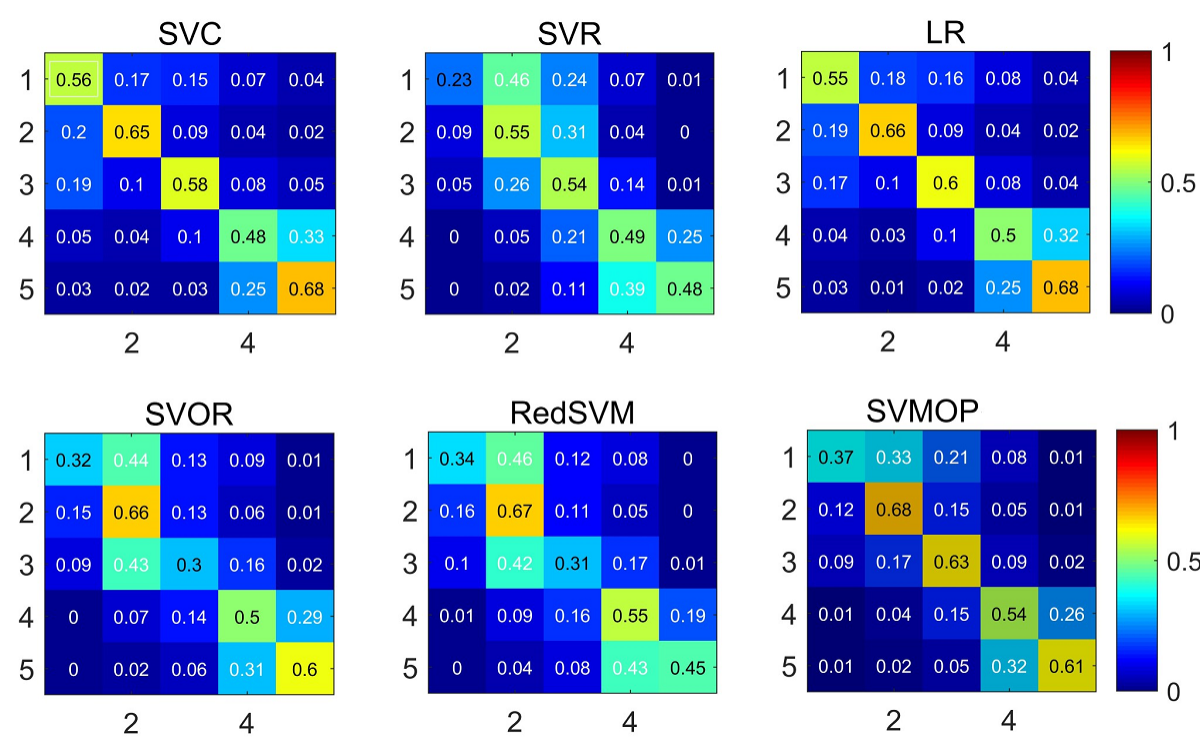
The confusion matrices of different models with the text, customized, and boosted feature set (T+C+B). RedSVM: reduction support vector machine; SVC: support vector classification; SVMOP: a combined support vector machine and ordinal partitioning scheme model; SVOR: support vector ordinal regression; SVR: support vector regression.

Additionally, the confusion matrices were used to further discuss the differences among the performance of different models. Each confusion matrix is generated by the corresponding model on feature set T+C+B. As shown in Figure 6, models that consider ordering information, such as SVOR, RedSVM, and SVMOP, misclassify the incorrectly labeled samples into the closest categories. For example, in the confusion matrix of SVMOP, the third cell of the third row shows that 63% of most (17%) misclassified instances fell into Category 2. In contrast to the confusion matrix of SVMOP, when looking at the third row of the confusion matrix of SVC, we find that most (19%) of the misclassified instances fall into Category 1. For this study, this example illustrates that the nonordering information methods, such as SVC and LR, can misclassify doctors having *neutral* performance levels into the *very unsatisfied* category. However, methods that consider ordering information of doctors’ performances, such as SVOR, RedSVM and SVMOP, are more likely to place misclassified neutral doctors into the *unsatisfied* category.

### Predictive Features Analysis

As for mining the most predictive features, (see equation 9), and after computing and sorting each feature, we find that 4 customized features are in the top 5 most predictive features, including F3, F4, F5, and F7. Feature F6 ranks 8th and feature F8 ranks 23rd. In other words, most of the customized features play the most predictive roles in DPE, which is consistent with our numerical results. In view of the most predictive text features, we find that the features that contribute most to the model decision are the polite expressions like: “不客气” (“You’re welcome”), “谢谢您” (“Thank you”), “很乐意帮助您” (“Glad to help you”) and sensitive words such as “好评” (“good rating”), “态度”(“attitude”), “五星”(“five-star”). Some words like “禁忌辛辣食物” (“avoid spicy foods”), “对身体有害” (“bad for health”) are helpful, and indicates that the doctor is explaining some issues in more detail. These features cannot guide in questionnaire design but are beneficial for platform building and optimization.

## Discussion

### Principal Results

Statistically, 36% of data on doctor/patient communication platforms has been labeled by patients, showing that 64% of the clinical data lack evaluation. Considering that doctors’ performance could affect patient satisfaction, we take the DPE task as an ordinal regression problem, ensuring the automatically predicted labels are as close as possible to the true ones. The OR-DPE, SVMOP model with revised prediction is applied as the core model, and the metrics of MAE, MSE, PAcc, and SVMOP models with feature set T+C+B could achieve state-of-the-art performance. Compared with other auto-evaluation models, SVMOP improves MAE by 0.1, MSE by 0.5, and PAcc by 5%. The customized features improve MAE by 0.1, MSE by 0.2, and PAcc by 3%. Additionally, with the boosting technique, the performance of SVMOP can be further improved. Furthermore, based on OR-DPE model, predictive features like polite expressions and sentiment words can also be mined, which can be used to guide the development of mHealth platforms.

### Comparison with Prior Work

The experiments conducted on real data have validated the effectiveness of SVMOP. Because of the noise in the real data, we continue to experiment on benchmark OR datasets [19] in a precisely controlled environment. The datasets can be downloaded from the public website. In this experiment, we compare our model with all the baselines mentioned in the paper. The details about the benchmark datasets and the results are shown in Tables 4 and 5.

We find that SVMOP always performs better than other baselines on MAE, MSE, and PAcc. The results verify the effectiveness of SVMOP on clean data. Therefore, the good results benefit from the SVMOP model but not the experimental data about DPE, which further demonstrates the correctness of choosing SVMOP as the core model of OR-DPE.

### Limitations

Although this study has solved the problem of doctors’ auto-evaluation on doctor/patient communication platforms by the ordinal regression approach, there are limitations. Firstly, the definition of a good consult here is related to user satisfaction, not to medical accuracy or clinical utility. A good doctor seems to be a likable one, but a likable one may make incorrect medical decisions. Secondly, Farmer et al [40] point out that doctors’ work should be evaluated by multiple complex professional factors. In other words, a good consult is not only related to patients but also to many other factors. One way to handle this issue is to multisource feedback [41], which is called 360-degree evaluation in which key performance behaviors are simultaneously rated by peers, patients, and coworkers. Considering the characteristics of doctor/patient communication platforms, peer evaluation can be achieved by questionnaires, and the predictive features generated by the OR-DPE model may, in turn guide the questionnaire design.

### Conclusions

The authors are the first to conceptualize the problem of DPE as an ordinal regression task and develop an OR-DPE model to address it. Apart from the basic text features, we use eight customized features suggested by domain experts as important features to improve model performance. Furthermore, we applied GBDT to boost the 8 customized features. Additionally, we proposed a new model called SVMOP which has a reasonable and effective prediction function. Experiments show that the performance of SVMOP is better than many other machine learning methods on MAE, MSE, and PAcc. In summary, with the OR-DPE model, the mHealth platform could not only make an auto-evaluation of online doctors’ performance but also mine the most effective features which can then be further applied to guide the promotion of doctors and platforms. In the future, we hope our model can also be explored and applied to other medical service-oriented issues in medical education.

**Table 4:** Benchmark datasets. “#ins” is the number of instances. “#fea” is the number of features. “#class” is the number of classes.

Datasets	#ins	#fea	#class
housing-5	10120	14	5
machine-5	4180	7	5
abalone-5	83540	11	5
housing-10	10120	14	10
machine-10	4180	7	10
abalone-10	83540	11	10

**Table 5:** The mean absolute error (MAE), mean standard error (MSE), and pairwise accuracy (PAcc) performances of different models on benchmark datasets. The best result is indicated by a footnote.

Datasets		SVC^a^	SVR^b^	LR^c^	SVOR^d^	RedSVM^e^	SVMOP^f^
**Mean absolute error (MAE)**							
	housing-5	0.517	0.454	0.435	0.398	0.403	0.366^g^
	machine-5	0.606	0.550	0.451	0.390	0.424	0.369^g^
	abalone-5	0.798	0.712	0.700	0.683	0.675	0.648^g^
	housing-10	1.513	0.962	0.999	0.859	0.848	0.757^g^
	machine-10	1.425	1.151	0.986	0.935	0.927	0.841^g^
	abalone-10	1.959	1.451	1.557	1.435	1.434	1.391^g^
**Mean standard error (MSE)**							
	housing-5	0.665	0.545	0.612	0.494	0.524	0.446^g^
	machine-5	0.994	0.634	0.648	0.469	0.505	0.429^g^
	abalone-5	1.450	0.992	1.244	1.042	0.991	0.962^g^
	Housing-10	4.564	1.858	2.560	1.694	1.642	1.453^g^
	machine-10	3.998	2.487	2.277	1.786	1.720	1.547^g^
	abalone-10	7.222	3.703	5.091	3.586	3.783	3.635^g^
**Pairwise accuracy (PAcc)**							
	housing-5	0.614	0.638	0.658	0.663	0.659	0.676^g^
	machine-5	0.602	0.604	0.652	0.666	0.655	0.680^g^
	abalone-5	0.547	0.553	0.584	0.584	0.577	0.589^g^
	Housing-10	0.552	0.623	0.609	0.635	0.637	0.642^g^
	machine-10	0.488	0.562	0.597	0.601	0.599	0.612^g^
	abalone-10	0.514	0.568	0.566	0.565	0.568	0.569^g^

^a^SVC: support vector classification.

^b^SVR: support vector regression.

^c^LR: logistic regression.

^d^SVOR: support vector ordinal regression.

^e^RedSVM: reduction support vector machine.

^f^SVMOP: a combined support vector machine and ordinal partitioning scheme model.

^g^Best result.

## Abbreviations

BOW: bag of words
CWS: Chinese word segmentation
DCD: Dual Coordinate Descent algorithm
DPE: doctor performance evaluation
GBDT: Gradient Boosting Decision Tree
LR: logistic regression
MAE: mean absolute error
mHealth: mobile health
MSE: mean square error
OR: ordinal regression
OR-DPE: ordinal regression for doctor performance evaluation
PAcc: pairwise accuracy
RedSVM: reduction support vector machine
SVC: support vector classification
SVM: support vector machine
SVMOP: a combined support vector machine and ordinal partitioning scheme model
SVOR: support vector ordinal regression
SVR: support vector regression
T: text features
T+C: text and customizable features
T+C+B: text, customizable, and boosted features
TF-IDF: term frequency inverted document frequency
